# Fiber-Optic Surface Temperature Sensor Based on Modal Interference

**DOI:** 10.3390/s16081189

**Published:** 2016-07-28

**Authors:** Frédéric Musin, Patrice Mégret, Marc Wuilpart

**Affiliations:** Electromagnetism & Telecom Department, University of Mons, Boulevard Dolez 31, Mons B-7000, Belgium; patrice.megret@umons.ac.be (P.M.); marc.wuilpart@umons.ac.be (M.W.)

**Keywords:** temperature, sensor, surface thermometry, fiber optic, interferometer, few-mode

## Abstract

Spatially-integrated surface temperature sensing is highly useful when it comes to controlling processes, detecting hazardous conditions or monitoring the health and safety of equipment and people. Fiber-optic sensing based on modal interference has shown great sensitivity to temperature variation, by means of cost-effective image-processing of few-mode interference patterns. New developments in the field of sensor configuration, as described in this paper, include an innovative cooling and heating phase discrimination functionality and more precise measurements, based entirely on the image processing of interference patterns. The proposed technique was applied to the measurement of the integrated surface temperature of a hollow cylinder and compared with a conventional measurement system, consisting of an infrared camera and precision temperature probe. As a result, the optical technique is in line with the reference system. Compared with conventional surface temperature probes, the optical technique has the following advantages: low heat capacity temperature measurement errors, easier spatial deployment, and replacement of multiple angle infrared camera shooting and the continuous monitoring of surfaces that are not visually accessible.

## 1. Introduction

Surface temperature measurements are intrinsically complex, due to the fact that the thermometer cannot be immersed. Moreover, except in adiabatic conditions, applying a sensor to the surface of an object inevitably disturbs its thermal equilibrium with the environment. Radiating techniques, like those used in infrared cameras, can sense an object remotely without disturbing the thermal exchanges. Nevertheless, they require precise knowledge of the emissivity of the material, the absence of external radiation perturbation, such as reflections and visual contact with the target. Moreover, they are not cost-effective if continuous monitoring is required. In this study, we will introduce an innovative contact surface temperature sensing technique, which uses fiber optic technology. Due to its small dimensions in the 100 µm range, fiber optic is a good candidate for close direct contact sensing of objects.

In this paper, fiber-optic sensing is presented as a solution for temperature variation sensing integrated over a surface. The concept of integrated measurements is defined here as the sum of all temperature variations that occur along the sensing fiber. Based on modal interference, the proposed technique relies on coherent light phase sensitivity to thermal solicitation. Historically seen as a disadvantage of fiber optic telecommunication, intermodal interference has been extensively studied from the modal noise point of view [1,2,3]. Previous works on temperature sensing mainly focused on intermodal interference processing by 1-D sensors, such as photodiodes [4,5,6,7,8] and CCD (Charge Couple Devices) arrays [9,10]. The self-calibrating capability of the system proposed in [11] is of particular interest. Sweeping wavelength approaches [12,13,14] introduce solutions for interferometric absolute temperature measurement. They include variable wavelength sources and optical spectrum analyzers in order to detect dips within the fiber spectral attenuation with respect to temperature. The advantage of these approaches relies on the ability to measure absolute temperature and to be sensitive within only a given section of the fiber thanks to the combined use of multimode and single mode fiber. Nevertheless, due to the wavelength detection principle, they lead to a higher cost compared to fixed wavelength interferometric techniques.

A new image-processing fiber optic interferometric intermodal temperature sensing technique was first introduced by the authors [15,16] in 2011. When applied to underground power cables, the solution enabled the continuous integrated monitoring of the infrastructure temperature so that any local overheating of a cable joint between 20 °C and 60 °C could be quantified with an accuracy of 1 K [17]. In order to determine the heating and cooling phase, a cyclically-heated fiber section was used [18]. The fields of application of this solution are either linear infrastructure (1D systems) monitoring, surface (2D systems) temperature sensing as proposed in this paper and 3D monitoring, such as gas or complex volume sensing.

Compared with other fiber-optic temperature sensors, based on the principles of Raman and Brillouin, which require expensive devices, the proposed intermodal interferometric technique only requires cost effective equipment such as fixed wavelength sources and common imaging devices. The sensitivity to temperature variation measurement is proportional to the fiber length [15,16,17,18] and can reach the mK range for a fiber length of a few hundred meters of G.652 fiber. The major drawback of the interferometric intermodal technique is its self-referenced nature, which makes it more suitable for measuring temperature deviations from an initial condition. Nevertheless, thanks to efficient image processing, the technique has been successfully applied to underground power cables and cable joints for absolute temperature monitoring [17] while limiting the time drift over several weeks. It is also worth noting that, for rate-of -rise heat sensing (an important group of sensors for fire safety applications), absolute temperature measurement is not required and recalibration is not necessary in the short term.

The purpose of this article is to propose an innovative integrated temperature surface sensor based on fiber optic modal interference. Compared with previous works, the technique has been enhanced by a new cooling/heating phase discrimination. This functionality was previously made possible by means of a complex cyclically heating and cooling device [18]. This complex equipment has been replaced by proper image processing, which is applied in specific optical modes and filtering conditions. The accuracy of the technique has also been improved by means of new image-processing for the interference pattern produced by a specific modal configuration. The performance of the proposed sensor is compared with conventional techniques (use of surface probes and an infrared camera). The hollow cylinder problem is used to illustrate the proposed method. The setup is designed in order to be strain insensitive so that temperature effect only is considered in this paper. Compared with the conventional technique, the fiber optic device can replace multi-angle infrared camera images making it possible to observe visually masked and large objects. Finally, another exclusive advantage of fiber, compared with conventional techniques, is that it can be extended to include volume temperature and gas temperature sensing, due to its easy spatial deployment.

## 2. Fiber Optic Intermodal Interferometer

### 2.1. Basic Principle

**Figure 1:**
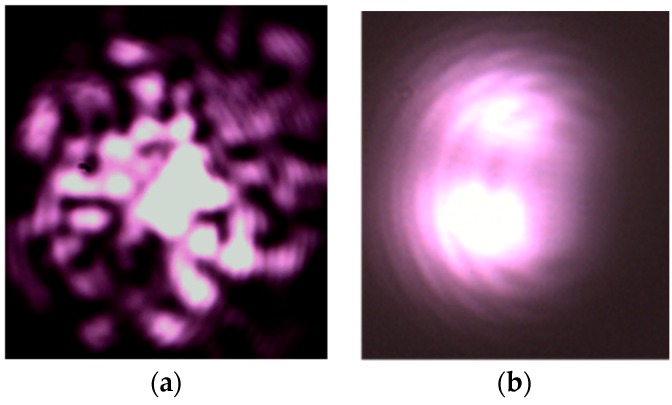
Example of the multi-mode interference pattern (**a**); and the few-mode interference pattern (**b**).

A variation in temperature induces a refractive index variation and elongation of the fiber. This results in changes to the propagation constant and fiber length. When stimulated with coherent light at one end of a multimode configuration, the fiber produces an intermodal interference pattern at the other end of the fiber, as illustrated in Figure 1. This pattern consists of moving spots that can be captured by a CCD or CMOS (complementary metal oxide semiconductor) camera. In multimodal conditions, it has been demonstrated that the spots average contour velocity of the interference pattern such as the one illustrated in Figure 1a is linked to temperature variation [15,16]. Figure 1a corresponds to a number of modes larger than 100. Using a few-mode configuration obtained by modal filtering, the interference pattern is only composed of one or two lobes that are easy to process (see Figure 1b) [18,19]. The number of modes interfering in the pattern depicted in Figure 1b is lower than 40. The continuous computation of the center of mass of the pattern results in a measurement of the pattern modification. It has been shown in [18] that this indicator is linearly linked to the temperature deviation integrated along the fiber. The “deviation” is taken from an initial condition of the integrated temperature measured along the fiber.

**Figure 2:**
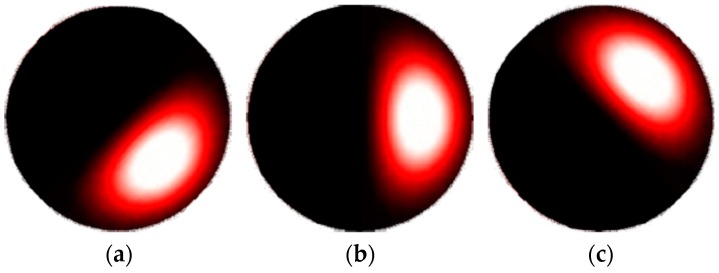
Rotating lobe pattern sequence (**a**), (**b**) and (**c**).

**Figure 3:**
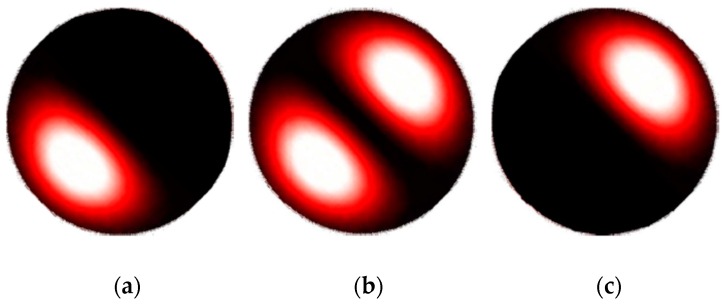
Pulsating lobe pattern sequence (**a**), (**b**) and (**c**).

The dynamics of the lobes of the few-mode pattern are illustrated in Figure 2 and Figure 3. As shown in [18,19], a single rotating lobe can be obtained by carefully selecting modes and controlling the phase shift between odd and even fields. For example, Figure 2 shows the pattern obtained from a combination of even and odd LP01 modes in phase and even and odd LP11 modes $\frac{\pi }{2}$ shifted. A change in the temperature of the fiber causes a rotation. As another example, Figure 3 shows the pulsation of one lobe obtained by the combination of LP_01_ and LP_11_ whose odd contributions are in phase. These conditions result in a pulsating lobe pattern with respect to the temperature variation of the fiber.

### 2.2. Heating-Cooling Phase Discrimination

The discrimination between a heating and a cooling phase, as described in [18], were based on the use of a cyclically calibrating and heated fiber section. This device produced an oscillating phase solicitation, which was amplified when the calibrating fiber and sensing fiber were being heated or cooled together and attenuated when the thermal solicitation was in opposition. In this paper, we propose to replace this cumbersome approach by processing the trajectory in the appropriate way, using the rotating lobe. In this context, the simple rotating pattern shown in Figure 2 has several advantages compared with the pulsating case of Figure 3.

**Figure 4:**
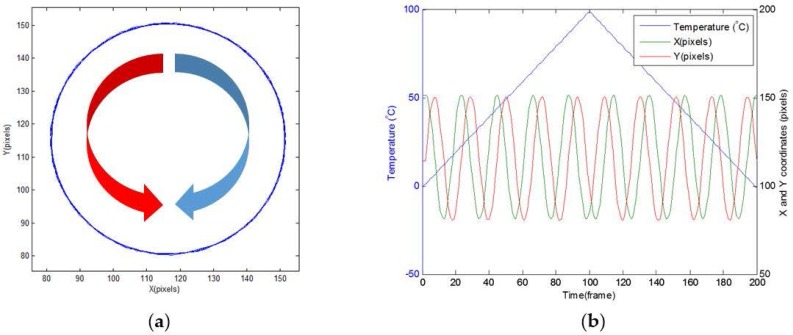
Center of mass calculation: rotating pattern in the counterclockwise direction for heating and in the clockwise direction for cooling (**a**), and coordinates along the X and Y axes of the center of mass (**b**).

For a single lobe rotating configuration, Figure 4a shows the simulated trajectory of the center of mass of the interference pattern produced by heating one meter of G.652 fiber from 0 °C to 100 °C and then cooling it from 100 °C to 0 °C. Phase conditions are: LP_01_ modes (even and odd in phase) and LP_11_ modes (even and odd, $\frac{\pi }{2}$ shifted). Figure 4b shows the development of the *X* and *Y* components of the center of mass of the interference pattern, in terms of temperature variation. *X* and *Y* correspond to the axes of the plane defined by the CMOS sensor in the camera. Their position is expressed in pixels for both *X* and *Y* coordinates. The trajectory followed by the center of mass of the pattern is a circle, whose *X* and *Y* coordinates are expressed by:

(1) $$\begin{array}{l} X(\theta )=r\cdot \cos(\omega_{\theta }L\theta +\phi_{0})\\ Y(\theta )=r\cdot \sin(\omega_{\theta }L\theta +\phi_{0}) \end{array}$$

with θ is the temperature, $\omega_{\theta }$ the pulsation related to the speed at which the circle is drawn with temperature, r the radius of the circle and L the length of the fiber subject to temperature variation. The dependency of the coordinates to the length of the fiber *L* is justified by the spatial integration capability of the sensor, as described in [20].

The transition from a heating phase to a cooling phase is observed in Figure 4b, by means of a U-turn of 180° of the trajectory of the center of mass at frame 100 corresponding to 100 °C. The red arrow in Figure 4a shows a heating phase, while the blue arrow shows a cooling phase. The direction of the rotation is also observed in Figure 4b: a heating phase, corresponding to a counterclockwise rotation, is characterized by an advance in phase of the *X* coordinate in relation to the *Y* coordinate; a cooling phase, corresponding to a clockwise rotation is characterized by an advance of phase of the *Y* coordinates. From the previous analysis, it appears that the sign of the temperature variation can be unambiguously deduced from the direction of rotation of the single rotating lobe pattern. The $\omega_{\theta }$ pulsation of Equation (1) is estimated by simulations at 300 mrad/(K∙m). This pulsation is in line with several other works on the subject [6,21,22,23].

**Figure 5:**
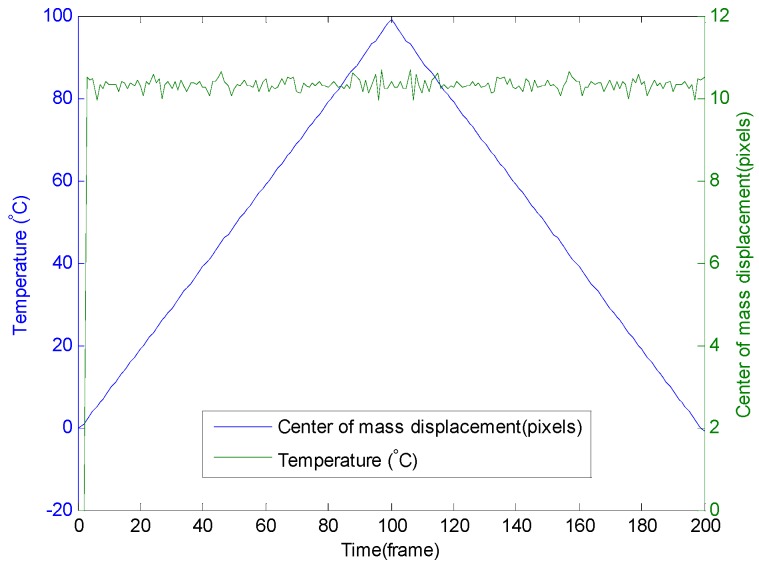
Displacement of the center of gravity of the interference pattern with the temperature variation from 0 °C to 100 °C and then from 100 °C to 0 °C.

The displacement of the center of mass and the temperature are given in Figure 5 for the same conditions of simulation. One can observe that the displacement (in pixels) is constant with the temperature variation at 10.3 ± 0.3 pixels/(K∙m) for a projection screen with a size of 220 × 220 pixels. The variation of 0.3 pixels/(K∙m) is due to the uncertainty over the gravity center position. The core radius of the fiber (4.5 µm) corresponds to 110 pixels in our simulations.

**Figure 6:**
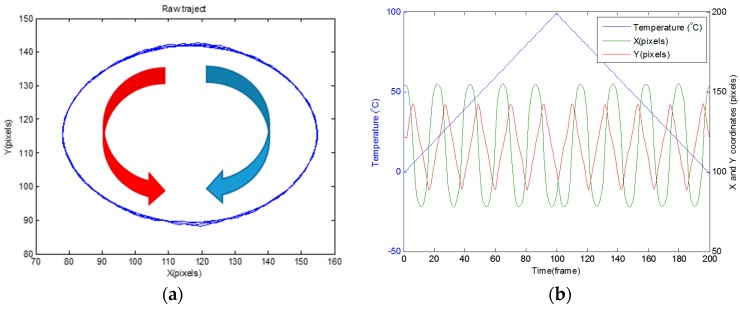
Center of mass calculation: elliptical rotating pattern in the counterclockwise direction for heating and in the clockwise direction for cooling (**a**), and coordinates along the X and Y axes of the center of mass (**b**).

When rotating and pulsating effects are both present, the dynamics between modes produces an elliptical pattern as illustrated in Figure 6 by simulation with the following conditions: LP_01_ modes (even and odd in phase) and LP_11_ modes (even and odd, $\frac{\pi }{3}$ shifted). The coordinates are:

(2) $$\begin{array}{l} X(\theta )=R\cdot \cos(\omega_{\theta }Lf(\theta )+\phi_{0})\\ Y(\theta )=r\cdot \sin(\omega_{\theta }Lf(\theta )+\phi_{0}) \end{array}$$

with θ is the temperature, $\omega_{\theta }$ the pulsation related to the speed at which the ellipse is drawn with temperature, R and r are the radii of the ellipse, f is a function of θ, and L is the length of the fiber.

**Figure 7:**
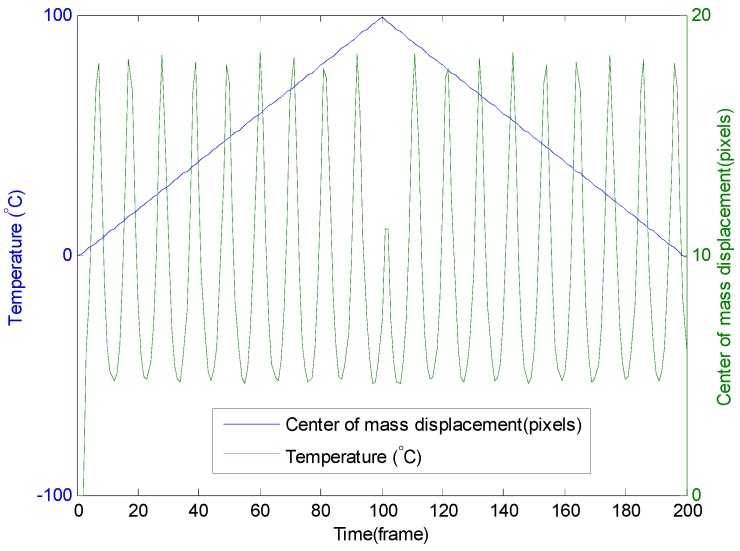
Displacement of the center of gravity of the interference pattern with the temperature variation from 0 °C to 100 °C and then from 100 °C to 0 °C.

The $\omega_{\theta }$ coefficient remains the same as for the circular trajectory conditions (300 mrad/(K∙m)). Nevertheless, there is a difference in the elliptical trajectory. As illustrated in Figure 7, at a constant temperature variation rate, the velocity of the center of mass is no longer constant. The speed of the center of mass near the short radius of the ellipse is higher than near the long radius. From the simulations results, the displacement of the center of mass varies between 5 and 18 pixels/°C per meter of fiber. This relation between the temperature and the displacement of the center of mass for an elliptical trajectory is symbolized by function f in Equation (2).

The spatially integrated temperature deviation of the fiber can be retrieved from the trajectory length with a 10.3 pixels/(K∙m) sensitivity coefficient. For the case of the circular trajectory and constant displacement versus temperature variation, temperature variation is simply proportional to the speed of the center of gravity of the interference pattern. For the elliptical trajectory, the relation is no longer linear. Methods used in [17,18] include the averaging of the displacement among at least one period. This limitation does not allow this previous method to be applied accurately to variations lower than 10 K for 1 m of fiber.

In this paper, we propose to use the phase of the *X* or *Y* coordinates components in order to achieve better accuracy for slow varying temperature schemes (<1 K for one meter of fiber). As observed in Equation (1) for a circular trajectory and on Figure 4b, the phase of the coordinate signals is linearly dependent to temperature. Temperature can, thus, be retrieved from the *X* coordinate signal thanks to Equation (3).

(3) $$\Delta \theta_{fiber}=\frac{1}{\omega_{\theta }L}\Delta \phi (X)$$

with $∆\theta_{fiber}$ the average temperature variation of the fiber, ∆φ(X) the phase variation observed. Heating and cooling phase are discriminated by processing the dephasing between *X* and *Y* coordinates signals. In practice, the ideal case of a circular trajectory is difficult to obtain because the dephasing between odd and even mode does not need to be exactly equal to $\frac{\pi }{2}$. An elliptical trajectory is, therefore, more likely but, in this case, the phase is no longer linear with the temperature, as illustrated in Figure 6b. For this particular set of simulation, the *Y* coordinate evolution clearly indicates that the phase does not show a linear behavior. This issue is resolved by extracting the fundamental harmonic of the coordinates. Thanks to this process, the *X* and *Y* coordinates from the elliptical trajectory, as shown in Figure 6b, are converted, in the ideal case of the circular trajectory of Figure 4b with pure sinusoidal signals (with different amplitude for *X* and *Y* coordinates), and Equation (3) remains applicable. In an experimental context, the best coordinate (*X* or *Y*) will be chosen for processing. The selection criteria will be the orientation along the long radius of the ellipse. It should be noted that this method is insensitive to the variation of the radii of the elliptical trajectory, which may vary in time, due to camera sensitivity variations.

**Figure 8:**
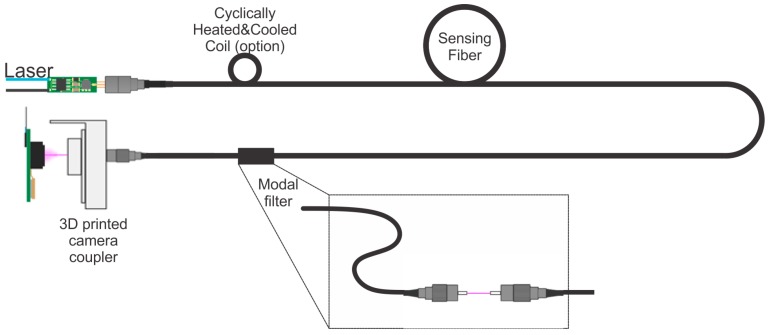
Optical setup.

In practice, the optical setup of the new configuration described in Figure 8 consists of a 100 mW 855 nm distributed feedback laser (EAGLEYARD PHOTONICS, Berlin, Germany), a G.652D sensing fiber, a modal filter and a five-megapixel camera (TERASIC, Hsin Chu, Taiwan), which is used to process the interference pattern. G.652D fiber characteristics and silica thermal expansion coefficient are presented in Table 1.

The modal filter consists of a fixed bend with a length of 20 cm and a space between two FC/APC connectors. The connector spacing makes it possible to select low-order modes and, thus, ensure a one-lobe condition. The fixed bend induces two phenomena [24]:

- A longitudinal stress stretching the fiber layers on the outside of the bend and compressing the layers on the inner side. This perturbation cannot induce coupling between modes of the same order.
- A compressive stress from the outer layers on the inner ones. This perturbation produces the same effect as birefringence: a detuning of the even and odd modes along the bend inducing a dephasing between modes.

Modal filters are sensitive to environment perturbations such as bending, strain, or temperature variation. This is why this device must be tightly fastened. The temperature sensitivity of the modal filter has been measured at 200 mrad/K. Both the connector spacing and fixed bend contribute to a trajectory that is easy to process, since they generate a single lobe pattern, which moves along an elliptical trajectory. A temperature variation lower than 1 K per meter of fiber is possible with this innovative approach. In the following sections, our method is applied to surface temperature sensing.

## 3. Integrated Surface Temperature Sensing with Fiber Optic

Integrated surface temperature sensing ideally requires the temperature to be measured at every point on a given surface. In practice, this continuous approach is replaced by discrete temperature measurements on a limited number of sub-surfaces. The surface temperature is then estimated using the average of these temperatures weighted by the area of each sub-surface. This method usually includes either infrared cameras or conventional surface temperature probes. Best practices [25] for temperature sensing include the following requirements, in order to obtain the most precise measurement:

- In order to minimize immersion errors, the sensor is immersed in the boundary layer between the object being tested and its environment as closely as possible to the surface. Ideally, the sensor should be placed along an isotherm line due to the fact that the temperature needs to be constant along its sensing part, in order to avoid spatial averaging errors. With conventional surface probes, the immersion errors will be harnessed thanks to the tight fastening of the sensors to the cylinder, the use of a heat conducting grease for better contact with the surface and, if possible, a thermal insulation of the system and sensor. This insulation keeps the sensor at a temperature as close as possible to that of the object being studied. This is not always possible, due to constraints imposed by the process or environmental conditions (for example, high temperatures).
- In order to minimize heat capacity errors, the sensor must have the lowest possible heat capacity in relation to the heat capacity of the system being monitored. This is due to the fact that the sensor needs to acquire heat from the system, in order to reach the thermal equilibrium before a correct measurement can be taken.
- Even if the heat capacity of the sensor is low compared with that of the system, it will take some time for the sensor to heat due to its thermal inertia (settling time response). This phenomenon is usually quantified by a time constant. Most probes and assemblies have a time constant, which increases by the square of their diameter [25]. Lag error is another consequence of the settling time response. When the temperature of the system changes too quickly, equilibrium is never achieved with the sensor. This type of error will be harnessed by limiting the temperature change rate and using the smallest possible sensors.

These requirements highlight the advantages of the fiber-optic method. A G.652D fiber has a core diameter of 8.2 µm and has a cladding diameter of 125 µm, which is far smaller than conventional temperature probes which usually measure over than 1 mm in diameter. The ability of the fiber to be attached to usual volumes, such as plates and cylinders, is another advantage. Consequently, fiber-optic technology should perform better in terms of immersion and settling response errors. Nevertheless, glass materials presents a lower conductivity than for instance stainless steel probes and acts like a thermal insulating material. This drawback can be balanced by small dimensions of optical fiber. In order to thermally compare optical fibers with conventional stainless steel cased pt100 sensors it is required to introduce the concept of material diffusivity. The latter represents the ability of a material to conduct thermal energy relatively to its ability to store thermal energy. In other words, with high thermal diffusivity, heat moves rapidly through the material because the medium conducts heat quickly relative to its volumetric heat capacity. Thermal diffusivity is defined by Equation (4):

(4) $$\alpha =\frac{k}{\rho c_{p}}$$

with α the thermal diffusivity, k the thermal conductivity, ρ the density, and $c_{p}\;$the heat capacity at constant pressure. Values of 0.34 mm^2^/s and 3.35 mm^2^/s can be found, respectively, for glass and stainless steel. To complete the analysis, the dimensions and the mass of the probe must be taken into account. Heat will move faster to the core of the probe if the probe is lighter and thinner. From the flash method described in [26] used to retrieve the thermal diffusivity of a material of thickness L in adiabatic conditions we know that a heat pulse applied at one side of a material will significantly reach the other side after a duration expressed by Equation (5).

(5) $$\sigma =K\frac{L^{2}}{\alpha }$$

with σ the time required for the back surface to reach half of the maximum temperature rise, L the thickness of the material and K a constant. This duration is inversely proportional to the thermal diffusivity and proportional to the square of the thickness of the material. Comparing optical fibers (glass with a thickness of 62.5 µm) to a conventional probe (stainless leads with a thickness of 500 µm) leads to time response six times shorter for the optical fiber. These performances are comparable to heated wire temperature probes used for air temperature measurements.

The fiber optic intermodal interferometer described above spatially integrates measurement of the temperature deviation from an initial condition. This statement is expressed by Equation (6):

(6) $$\Delta \theta_{fiber}=\frac{1}{L}\int \Delta \theta (l)\cdot dl$$

with $∆\theta_{fiber}$ is the variation of the temperature integrated all along the optical fiber, L the length of the fiber, and l is the infinitesimal length of the fiber.

In order to stay as close as possible to the surface temperature measurements obtained using the optical fiber method, we propose the following method:

- The fiber is laid on the whole surface of the object so that its temperature is as close as possible to that of the monitored object, with minimal disturbance of the thermal equilibrium between the object and its environment. Thermal grease between the object and the fiber helps to reach this target.
- The fiber is attached in such a way that the thermal expansion or contraction of the observed object does not induce strain effect on the fiber. This condition is achieved thanks to an interlayer between the fiber and the object. Again, thermal grease between the object and the fiber is used.
- The spatial gradient of the temperature of the monitored surface (in K/m) multiplied by the inter-distance between two adjacent sections of the monitored fiber (in m) is several times lower than the required temperature accuracy (in K).

In these conditions we have:

(7) $$\Delta \theta_{surf}=\Delta \theta_{fiber}=\frac{1}{L}\int \Delta \theta (l)\cdot dl=\frac{1}{\omega_{\theta }L}\Delta \phi (F(X))$$

where *F*(*X*) represents the fundamental of *X*.

In conclusion, the temperature variation of a surface sufficiently covered by an optical fiber is close to the temperature variation spatially-integrated along the fiber.

## 4. Surface Temperature Experimental Setup

**Figure 9:**
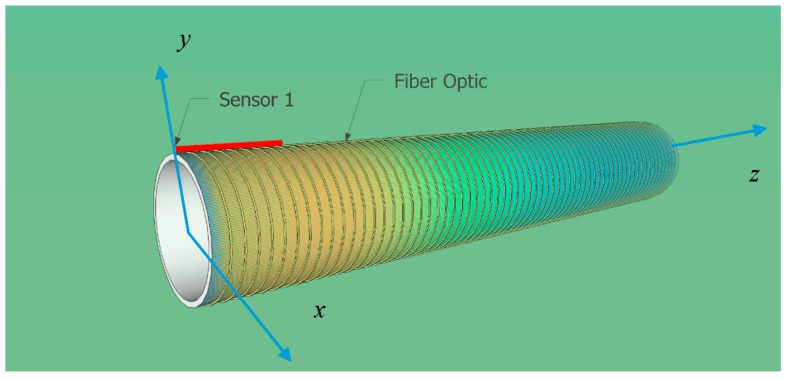
Calibrated infrared camera surface measurement technique.

In order to demonstrate the efficiency of the fiber optic surface temperature technique, the hollow cylinder case is shown in detail. The purpose of this experimental part is to demonstrate the ability of the fiber to measure surface temperatures. We compared the fiber optic measurement with a calibrated infrared camera method in the setup illustrated in Figure 9. The sampling rate is 1 sample/min. It should be noted that this setup is not adapted to evaluate the response time of the optical technique.

The setup includes a reference probe combined with an interrogator with an overall accuracy of ±0.05 °C (Sensor 1–Manufacturer and reference: TESTO, Lenzkirch, Germany—Model 735-2, with high precision pt100 probe). This sensor is attached to the cylinder with an intermediate thermal paste layer with a thickness of several hundreds of µm. The total uncertainty of this measuring system is lower than 0.5 K due to the fact that the trials are conducted at ambient temperature (∆θ = 5 K max), therefore, limiting immersion and radiation errors. The dominant part of the uncertainty comes from the settling response time estimated at 0.4 K and due to the time response of the probe (60 s) in the context of the experiments (variation in temperature of 0.4 K/min).

The thermal gradient is quantified using an infrared camera (Manufacturer and type: Fluke-Ti20, Dubai, UAE) with an absolute accuracy of 2 °C and a relative accuracy between two points of 0.1 °C. The surface temperature is computed from the average taken from a 160 × 120 temperature matrix extracted from the IR camera. Partial errors result from the following variables: emissivity, reflected ambient temperature, atmosphere temperature, and camera accuracy. Uncertainties due to atmosphere and radiation reflection from the environment are not considered in the estimation thanks to trials conducted at ambient temperature (∆θ = 5 K max) and the shielding of the working area. Emissivity and camera uncertainties are kept lower than 0.1 K thanks to the calibration with the reference probe at constant temperature (no settling response error). In conclusion, the total uncertainty of the surface temperature measurement including the precision probe and the infrared camera is 0.6 K.

**Figure 10:**
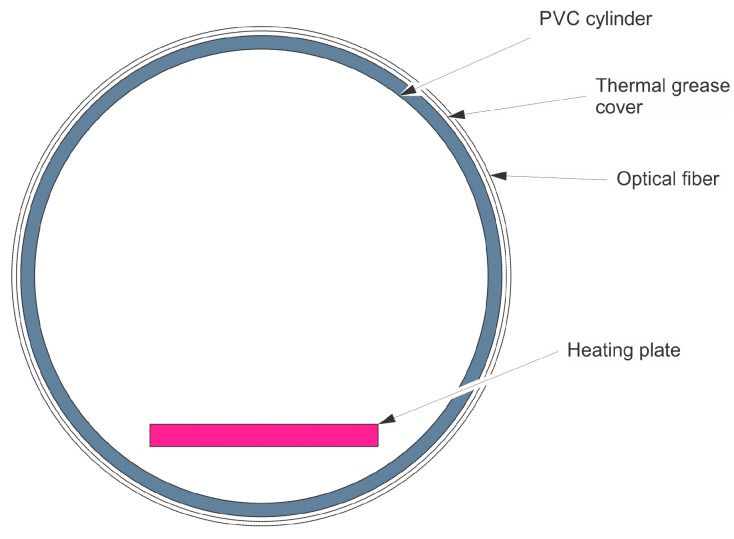
Cross-section of the setup at z = 20 mm.

The cylinder used in the experimental setup is made of PVC and has an external diameter of 50 mm, a thickness of 3 mm and a length of 300 mm. A heat source is placed inside the cylinder at one end. The heating device consists of an electrical resistance delivering a power of 3 W. A cross-section of the setup at z=20 mm is depicted in Figure 10.

Twenty meters of jacketed G.652D optical fiber with a diameter of 900 µm are used in this trial. The major part (19.5 m) of this fiber is laid on the cylinder. Two short sections of 25 cm connect the fiber laid on the cylinder to the interrogator. These short sections and the modal filter are kept at constant ambient temperature during the trials (temperature variation less than 1 K). Their contribution to the sensitivity of the system is negligible (maximum 500 mrad) compared to the response of the 19.5 m of fiber (12 rad/K). Thermal grease has been laid between the cylinder and the optical fiber. This thin layer of several hundreds of microns plays a dual role: thermal coupling between the cylinder and the optical fiber on the one hand and loose attachment of the fiber to the cylinder on the other hand. This setup prevents the fiber from undergoing the strain effect caused by cylinder thermal expansion and contraction. Due to the fact that PVC presents an expansion coefficient 100 times larger than optical fiber, this precaution is of high importance. This assumption is verified by comparing the temperature sensitivity coefficient obtained experimentally to the one obtained by simulations and bibliography. Similar sensitivity coefficients have been obtained for loose fiber and attached fiber with this method. Therefore, it asserted that the designed setup is not sensitive to the thermal expansion and contraction of the cylinder.

**Figure 11:**
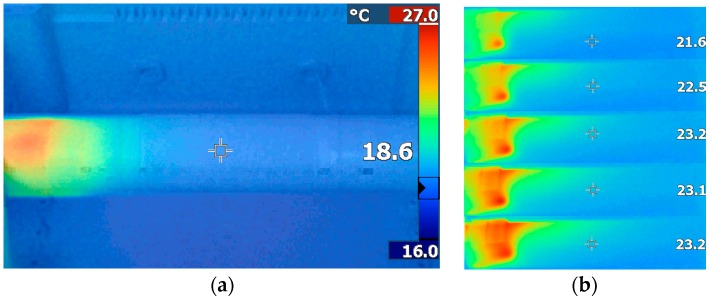
(**a**) The temperature gradient applied to the cylinder measured by an IR camera; and (**b**) from top to bottom: infrared pictures taken at one-minute intervals during a heating phase. Values to the right of the figure show the temperature targeted by the cross in the center of the figures.

The heating source was placed intentionally at one end of the cylinder and the other end was left exposed without insulation, in order to deploy a heat gradient in the cylinder axis direction. This temperature gradient is shown by the IR image in Figure 11a. The color bar on the right side of the figure shows the range (in this case, 16 °C–27 °C). The temperature of the point targeted by the central cross in the picture is measured by the camera (18.6 °C). Examples of infrared pictures are also shown in Figure 11b and were taken at a one-minute intervals during a heating phase of the cylinder.

The linear fiber optic sensor is implemented so that the temperature gradient in the direction perpendicular to its axis is small enough to identify a constant temperature between two successive loops. According to the infrared analysis, an inter-distance of 2.5 mm is necessary, in order to present a temperature difference of less than 0.1 °C between two passes of the sensor.

## 5. Results

**Figure 12:**
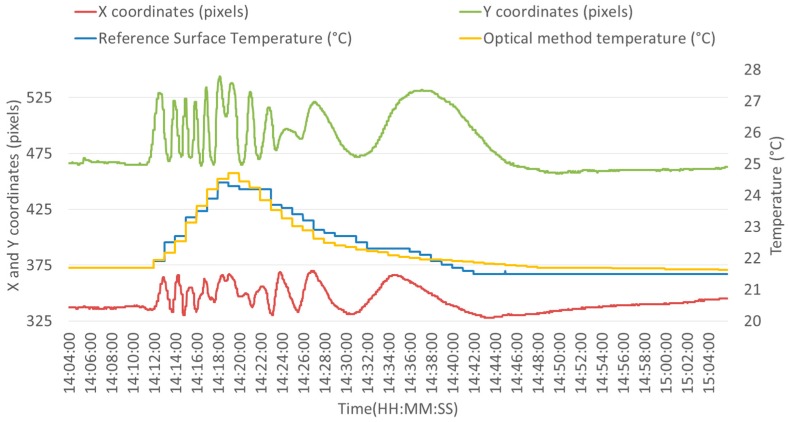
Fiber optic and reference temperatures. *X* and *Y* coordinates.

Optical fiber and infrared camera measurements are shown by the following lines and figures. During this experiment, the system was heated with a power of 3 W for approximately eight minutes and then naturally cooled down by the air. The surface temperature shown in blue in Figure 12 and measured by the reference infrared camera starts at 21.7 °C, peaks at 24.4 °C and finally cools down to 21.5 °C. The Y coordinate was chosen, in order to retrieve temperature information and is shown in green in Figure 12.

This behavior is in line with the theoretical development suggested in Section 2. An elliptical trajectory can be observed for the center of mass of the interference pattern. The direction of rotation along this trajectory is unambiguously linked to the heating or cooling phase. On the one hand, it can be seen that the *Y* coordinate is ahead of the *X* coordinate during the heating phase. On the other hand, it can also be observed that the *X* coordinate is ahead of the Y coordinate during the cooling phase. A U-turn can be observed between 14.18 and 14.20, which corresponds to the transition from the heating phase to the cooling phase.

The pulsation $\omega_{\theta }$ of Equation (3) is estimated at 600 ± 50 mrad/(K∙m). It is evaluated thanks to a calibration phase using a time-varying temperature kept homogeneous all along the optical fiber. The difference between this value and 300 mrad/(K∙m) observed in simulations is attributed to the different expansion coefficients and refractive index chosen for simulation and those of the sensing fiber used in the experiment. The uncertainty of the phase measurements is estimated at 1600 mrad. This represents in the context of this setup (20 m of fiber) an uncertainty of 0.15 K.

**Figure 13:**
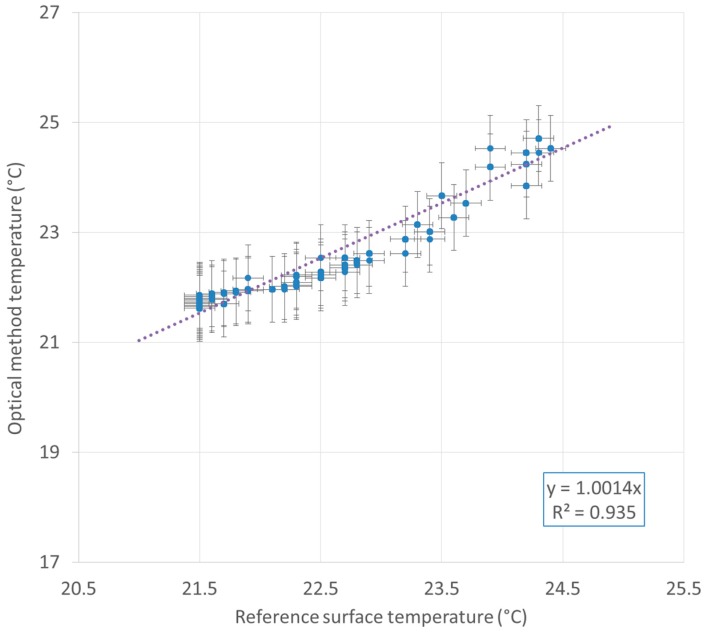
Optical method temperature versus reference surface temperature and linear regression.

For this trial, the maximum absolute difference between the optical fiber method and the infrared method is 0.63 K. The measurable range depends on fiber characteristics. The same 900 µm jacketed G.652D fiber was tested successfully in the 20 °C–90 °C range [17]. Figure 13 develops the temperature obtained by the optical method versus the reference surface temperature. A linear regression showing a determination coefficient *R^2^* of 0.935 has been determined. This experiment was reproduced five times at an interval of several days, leading to similar results and a sensitivity coefficient with a variation of less than 10%.

## 6. Materials and Methods

This article focuses on a method used to determine the integrated temperature variations of an optical fiber and includes the following stages:

- Selecting a few-mode optical fiber.
- Lighting one end of this optical fiber using a coherent light source.
- Determining the interference pattern of coherent light at the other end of the optical fiber.
- Selecting the maximum intensity at the other end of the optical fiber by spatially filtering the lighting used on the optical fiber.
- Determining the trajectory of the selected maximum intensity.
- Based on the determined trajectory of the selected maximum intensity, determining the temperature variation of the different mode optical fibers using the phase signal of the *X* or *Y* components of the center-of-mass.
- Determining signs of temperature variation from the phase lag between the *X* and *Y* components of the center-of-mass.

## 7. Conclusions

A fiber-optic surface temperature sensor is described in this paper. Intermodal interference patterns are processed by a camera, in order to extract the temperature deviation. If applied to a hollow cylinder with a temperature spatial gradient, the proposed device is comparable in terms of performance to a reference system consisting of an infrared camera and a precision contact thermometer. A maximum absolute difference of 0.63 K was reached between the temperature obtained by the optical method and the reference temperature. The contribution of this work mainly resides in the major improvement of the basic intermodal interferometric fiber optic temperature and its application to surface temperature sensing. This innovative technique makes it possible to measure the temperature variation of less than 1 K per meter of fiber, while the previous version of the technique was limited to 10 K per meter of fiber. The heating and cooling phase are identified by sharp mode filtering, which produces an interference pattern of only one lobe along an elliptical trajectory. The direction of the temperature variation is then deduced from the direction of the lobe rotation along the elliptical trajectory. The temperature difference spatially integrated along the fiber is derived from the phase of the *X* or *Y* coordinate of the center of mass of the various mode interference patterns. Compared with the conventional technique, the fiber optic device can replace multi-angle infrared camera images and allows visually-masked objects to be observed.

## Figures and Tables

**Table 1:** G.652D optical fiber characteristics and silica properties.

Characteristics	Value
Cladding diameter	125 µm ± 0.7 µm
Core diameter	8.2 µm
Cabled Cut-Off Wavelength	1260 nm
Refractive Index Profile	Step-index
Numerical aperture	0.14
Refractive index difference	0.36%
Silica thermal expansion coefficent	5 × 10^−6^ $K^{-1}$
